# Highly Deformed *o*‐Carborane Functionalised Non‐linear Polycyclic Aromatics with Exceptionally Long C−C Bonds

**DOI:** 10.1002/chem.202004517

**Published:** 2020-12-23

**Authors:** Adam V. Marsh, Mark Little, Nathan J. Cheetham, Matthew J. Dyson, Matthew Bidwell, Andrew J. P. White, Colin N. Warriner, Anthony C. Swain, Iain McCulloch, Paul N. Stavrinou, Martin Heeney

**Affiliations:** ^1^ Department of Chemistry and Centre for Processable Electronics Imperial College London London SW7 2AZ UK; ^2^ Department of Physics and Centre for Processable Electronics Imperial College London London SW7 2AZ UK; ^3^ Molecular Materials and Nanosystems and Institute for Complex Molecular Systems Eindhoven University of Technology 5600 MB Eindhoven Netherlands; ^4^ AWE Reading RG7 4PR UK; ^5^ Department of Chemistry Chemistry Research Laboratory University of Oxford Oxford OX1 3TA UK; ^6^ Department of Engineering Science University of Oxford Oxford OX1 3PJ UK

**Keywords:** AIE, carboranes, charge transfer, fluorescence, polycyclic aromatic hydrocarbons

## Abstract

The effect of substituting *o*‐carborane into the most sterically hindered positions of phenanthrene and benzo(k)tetraphene is reported. Synthesised via a Bull–Hutchings–Quayle benzannulation, the crystal structures of these non‐linear acenes exhibited the highest aromatic deformation parameters observed for any reported carborane compound to date, and among the largest carboranyl C−C bond length of all organo‐substituted *o*‐carboranes. Photoluminescence studies of these compounds demonstrated efficient intramolecular charge‐transfer, leading to aggregation induced emission properties. Additionally, an unusual low‐energy excimer was observed for the phenanthryl compound. These are two new members of the family of carborane‐functionalised non‐linear acenes, notable for their peculiar structures and multi‐luminescent properties.

## Graphical abstract

***o*** **‐Carborane‐functionalised** phenanthrene and benzo(k)tetraphene are reported in this work; two highly sterically hindered structures, with the latter displaying the largest aromatic deformation parameters of any organo‐substituted *o*‐carborane compound, and amongst the longest carboranyl C−C bond. Functionalisation of these polycyclic aromatic hydrocarbons induces stimuli‐responsive luminescence properties, including aggregation‐induced emission.

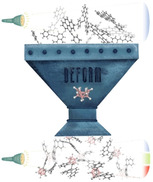

*O*‐carborane, an icosahedral compound of formula C_2_B_10_H_12_, is the most widely studied of the organoborane clusters. Following its classified origins,^[1]^ the first reports of *o*‐carborane in the open literature revealed its unusual properties, which have since been exploited for a range of materials science purposes.^[2]^ In recent years, attention has turned to the use of *o*‐carborane as a functional unit in organic semiconductors,^[3]^ with a focus on developing stimuli responsive luminescent materials.^[4]^

There are several intrinsic properties of *o*‐carborane which make it attractive for use in luminescence applications. Famously, carboranes are extraordinarily chemically and thermally stable,^[2]^ and confer some of this stability onto systems to which they are attached.^[5]^ This stability enhancement has been observed for organic electronic materials where large, oxidatively susceptible, π‐systems are prevalent.[^6^, ^7^, ^8^, ^9^] The bulkiness of the *o*‐carborane cage is also known to suppress concentration quenching in organic fluorophores.^[7]^ Furthermore, while *o*‐carborane itself is not photoluminescent, its presence can have a powerful positive effect on the solid state emissive properties of compounds onto which it is attached.^[10]^

When bonded directly to an aryl fluorophore, *o*‐carborane acts as an acceptor moiety, accommodating an excited charge from the aryl donor in the photoexcited state. This leads to the formation of an intramolecular charge‐transfer (ICT) state, in which the electron density is highly localised on the cage.[^11^, ^12^, ^13^, ^14^, ^15^, ^16^] Charge transfer has been shown to occur preferentially when the carboranyl carbon–carbon (C_C_−C_C_) bond is perpendicular to plane of the donor fluorophore and, structurally, this state is notable for an elongated carborane C_C_−C_C_ bond.^[17]^ In good solvents, these ICT states are very weakly emissive, as vibration of the C_C_−C_C_ bond acts as a non‐radiative decay pathway for the excited state.[^18^, ^19^, ^20^, ^21^, ^22^] Whenever molecular motion is restricted, however, carboranyl ICT states become more radiative. This often occurs upon aggregation, in a process known as aggregation induced emission (AIE).[^7^, ^17^, ^18^, ^20^, ^21^, ^22^, ^23^, ^24^, ^25^, ^26^, ^27^, ^28^, ^29^, ^30^, ^31^, ^32^, ^33^, ^34^, ^35^, ^36^, ^37^, ^38^, ^39^] *o*‐Carborane is therefore considered an ‘AIE‐active element block’.^[40]^

The AIE efficiency in carborane‐containing fluorophores is enhanced when the rotation of the carborane is structurally restricted around the ICT geometry (i.e., when the C_C_−C_C_ bond is approximately perpendicular to plane of the fluorophore). This has been achieved by introducing sterically large groups at the second carboranyl carbon atom, as well as incorporating the carborane into sterically demanding positions on the aromatic fluorophore.[^7^, ^41^, ^42^, ^43^] The resulting steric strain causes the C_C_−C_C_ bond to lie perpendicular to the plane of the fluorophore, accompanied by severe deformation of the aromatic backbone and a large increase in potential energy upon carborane rotation. In effect, the carborane becomes restricted in a potential energy well around a geometry favouring an ICT state. Using this design strategy, carboranyl fluorophores in an aggregated state have reached exceptional photoluminescent quantum yields (*φ*_PL_ >0.99).[^7^, ^42^, ^43^]

Our recent work introduced bis(phenyl‐*o*‐carborane)chrysene,^[23]^ a non‐linear acene molecule of this architecture. Carborane incorporation severely distorted the aromatic structure, and the presence of the bulky phenyl group yielded a rigid ground state with the C_C_−C_C_ bond perpendicular to the aromatic plane. The compound exhibited an array of photoluminescent properties, including a rare chrysene excimer not previously observed in solution, and AIE with good *φ*_PL_. This demonstrated that addition of *o*‐carborane induced both AIE and a variety of tuneable luminescent species in non‐linear acenes, suggesting the potential of such materials in fluorescent sensing applications.

In the present study, the family of *o*‐carborane‐functionalised non‐linear acenes is further developed by attaching phenyl‐*o*‐carborane into the most sterically demanding positions of phenanthrene and benzo(k)tetraphene (Figure 1). The resulting materials exhibit highly twisted aromatic cores with significant deformation of the carborane ring and contain some of the longest C_C_−C_C_ bonds reported to date. Both materials exhibit a range of interesting photophysical behaviours including efficient ICT, AIE, and, in the case of the phenanthryl compound an unusual low‐energy excimer.

**Figure 1:**
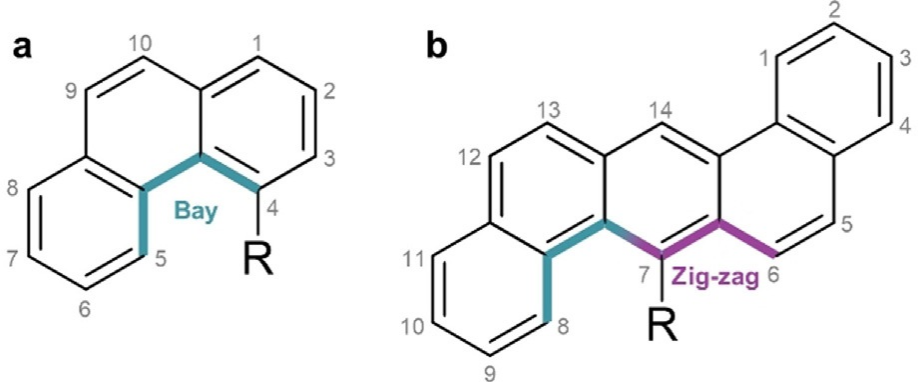
The structure of (a) phenanthrene substituted at the 4‐position, and (b) benzo(k)tetraphene substituted at the 7‐position, with polycyclic aromatic hydrocarbon edge nomenclature highlighted.^[44]^

Phenanthrene is the simplest non‐linear acene, with substituents at the C(4)‐position (in the ‘bay’ region) encountering strong steric repulsion from the C−H vertex at the C(5)‐position. For benzo(k)tetraphene, substituents at the C(7)‐position sit between both a bay, and a ‘zig‐zag’ region, experiencing steric repulsion from the C−H vertices in the C(8)‐ and C(6)‐positions, respectively. Incidentally, the same is true for the four‐ringed tetraphene; however, the asymmetry of the molecule creates a difficult synthetic challenge concerning C(7) substitution. Our strategy to the target materials 4‐(phenyl‐*o*‐carborane)phenanthrene (**1**) and 7‐(phenyl‐*o*‐carborane)benzo(k)tetraphene (**2**) is shown in Scheme 1, and relied upon the cycloaddition reaction with a substituted alkyne to form the carborane cluster.

**Scheme 1:**
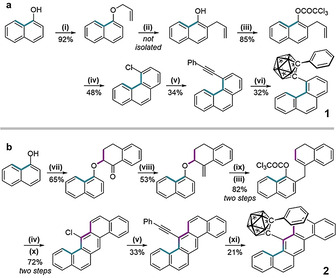
The synthesis of **1** and **2**. Reaction conditions: (i) allyl bromide, K_2_CO_3_, MeCN, rt, 12 h; (ii) neat, 210 °C, 2 h; (iii) trichloroacetylchloride, pyridine, ether, 0 °C, 2 h; (iv) CuCl, diglyme, reflux, 2 h; (v) phenylacetylene, Pd(PPh_3_)_2_Cl_2_, P(Cy)_3_, Cs_2_CO_3_, DMF, 110 °C, 24 h; (vi) B_10_H_14_, bmim(Cl), Tol, 110 °C, 48 h; (vii) 2‐bromo‐1‐tetralone, K_2_CO_3_, acetone, rt, 12 h; (viii) MePPh_3_Br, K^t^OBu, ether, 0 °C, 12 h; (ix) pyridine, 115 °C, 2 h; (x) DDQ, DCB, 150 °C, 1 h; (xi) B_10_H_12_(MeCN)_2_, AgNO_3_, Tol, 110 °C, 48 h.

The synthesis of **1** proceeded by the preparation of 4‐chlorophenanthrene via the Bull‐Hutchings‐Quayle (BHQ) reaction; a benzannulation from the trichloroacetate.^[45]^ Installation of the phenylacetylene group occurred through relatively mild Sonogashira conditions, aided by the activated nature of these aryl chlorides.^[45]^ Finally, the formation of **1** was achieved using an ionic‐liquid catalysed decaborane reaction^[46]^ in reasonable yield. The synthesis of **2** proceeded similarly, but with two extra steps; first, a Wittig reaction (step viii) to methylate the tetralone and, second, oxidative aromatisation (step *x*) of the ring system. Pure powders of the products were isolated and fully characterised, with NMR and high‐resolution mass spectrometry data presented in Figures S1–S8 of the Supporting Information. Recrystallisation of **1** and **2** from a biphasic solution of DCM and hexane yielded green and red single‐crystals, respectively, which were suitable for X‐ray diffraction analysis. The crystal structures of each are presented in Figure 2, with detailed crystallography data presented in Tables S1–2 of the Supporting Information.

**Figure 2:**
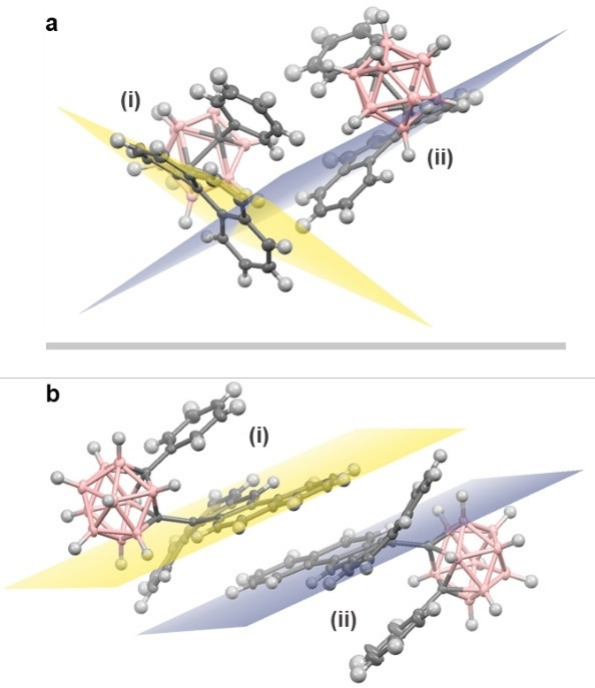
Asymmetric unit crystal structures of (a) **1**, and (b) **2**, with independent molecules labelled (i) and (ii). Approximate aromatic planes have been added to highlight deformation.^[47]^

**1** and **2** crystallised with two independent molecules in the asymmetric unit. In each case, the C_C_−C_C_ bond lies perpendicular to the aromatic plane because of the bulky phenyl substituent and its favourable π‐interactions with the aryl group. While **1** shows no evidence of intermolecular π–π stacking, **2** exhibits some degree of dimerisation, albeit with little spatial (and presumably orbital) overlap. Most strikingly, however, the ring system of both **1** and **2** are extremely distorted due to the incorporation of *o*‐carborane. This is exemplified by the aromatic deformation parameters and C_C_−C_C_ carborane bond lengths presented in Table 1.

**Table 1:** Aromatic deformation parameters and C_C_−C_C_ bond length for **1** and **2**.

Compound	α [°]	β [°]	θ [°]	C_C_−C_C_ Bond Length [Å]
**1** (i)	15.65	13.15	168.55	1.759(2)
**1** (ii)	14.77	10.88	169.27	1.769(2)
**2** (i)	22.82	12.93	166.34	1.829(3)
**2** (ii)	23.37	13.95	166.30	1.827(3)

As indicated by the large α and β angles (outlined in Figure S9), both independent molecules of **1** in the asymmetric unit contain *o*‐carborane moieties that are significantly raised above the aromatic plane. Similarly, the value of θ, which is the degree to which the carborane is tilted away from an ideal 180° rotation axis, demonstrates the carboranes are tilted toward the phenanthrene ring system, reflecting the favourable phenyl π‐stacking interactions.^[42]^ The C_C_−C_C_ bond lengths of both **1** molecules are also extremely elongated relative to that of *o*‐carborane (1.626 Å).^[48]^ These observations are also true for the independent molecules of **2**, but greater in magnitude. In fact, to the best of our knowledge, the aromatic deformation of **2** is the largest of any reported carboranyl compound (Table S3), and almost to the degree of 9,10‐bridged anthracenes.^[49]^ Similarly, the C_C_−C_C_ bond lengths of **2** are among the largest of all neutral carboranes substituted with aromatic groups;^[2]^ other substituents (e.g. thiocrown ethers and amino groups) can further increase the C_C_−C_C_ bond length of *o*‐carboranes.[^50^, ^51^]

The effects of the aromatic distortion on *o*‐carborane rotation, and the overall rigidity of the molecules, was investigated with a density functional theory (DFT) computational study, Figure S10. Rotation of the carboranes away from the ground state geometry leads to a significant increase in potential energy, particularly when rotation is directed towards the phenanthryl protons. Molecules will therefore demonstrate an especially strong tendency to adopt the perpendicular C_C_−C_C_ ground state geometry, thus aiding ICT state formation following excitation.

The effects of these structural constraints on the ultraviolet‐visible (UV/Vis) and photoluminescence emission (PL) properties of **1** and **2** were examined through a series of optical measurements, presented in Figure 3.

**Figure 3:**
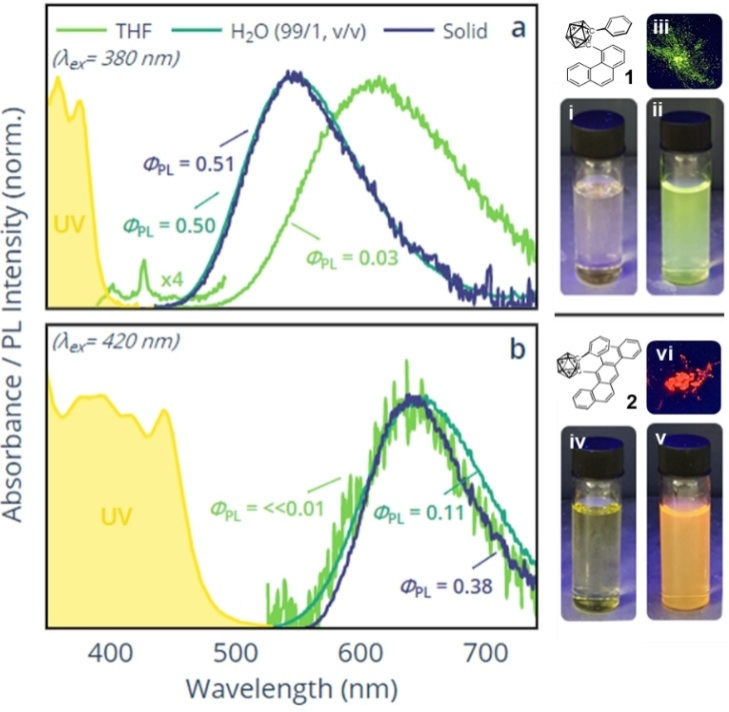
UV/Vis (THF) and peak‐normalised PL spectra measured in THF, H_2_O/THF (99/1, v/v) and the solid state for (a) **1**, and (b) **2**. Pictures of the UV (365 nm) irradiated samples measured in THF, H_2_O/THF (99/1, v/v) and the solid state are presented for **1** in (i), (ii), and (iii), respectively, and for **2** in (iv), (v), and (vi), respectively. Concentrations =10^−4^ m, except PL of the THF solution of **2** where the concentration =10^−3^ m owing to its weak luminescence.

Referring to Figure 3 a, the solutions of **1** in THF, while weakly emissive, displayed a broad emission feature (*λ*_max_=615 nm, *φ*_PL_=0.03) that is strongly red‐shifted relative to the absorbance edge. An even weaker and higher‐energy feature, exhibiting vibronic structure, was also detected at *λ*_max_=426 nm. In H_2_O/THF (99/1, v/v) and the solid‐state, the molecules of **1** aggregate and the spectra become dominated by, what appears to be a second emissive species, occurring at higher energy (*λ*_max_=548 nm) and accompanied by a significant increase in emission efficiency (*φ*_PL_=0.50 and 0.51, respectively). In contrast, and under the same conditions, the equivalent spectra for **2** show only broad emission features, centred around *λ*_max_=640 nm (Figure 3 b). Solutions of **2** in THF were again found to exhibit inferior PL quantum yields (*φ*_PL_ ≪0.01). However, in an aggregated state, from either the H_2_O/THF solutions or in the solid‐state, the emission efficiency once again dramatically increased (*φ*_PL_=0.11 and 0.38, respectively). Both **1** and **2** clearly demonstrate AIE properties.

A solvatochromic study was employed to probe the nature of the emissive species and is conveniently presented in the form of a Lippert–Mataga plot in Figure S11. The Stokes shift of each emissive species is compared, as a function of solvent orientation polarizability, with a positive linear correlation indicative of an excited state with a significant dipole moment.^[52]^ The Stokes shift of the short wavelength emission (*λ*_max_=426 nm) of **1** was found to be independent of solvent and, suggesting a localised emissive species (LE), is attributed to the phenanthrene ring system. The long‐wavelength emissive features of **1** and **2** are, however, solvent‐dependent, implying that these states have a delocalised character. Such delocalised states can be intramolecular (e.g. ICT) or intermolecular (e.g. excimeric) in nature. Further concentration and H_2_O/THF v/v solvent dependent optical studies were undertaken to confirm their origin and presented in Figures 4 and S12.

**Figure 4:**
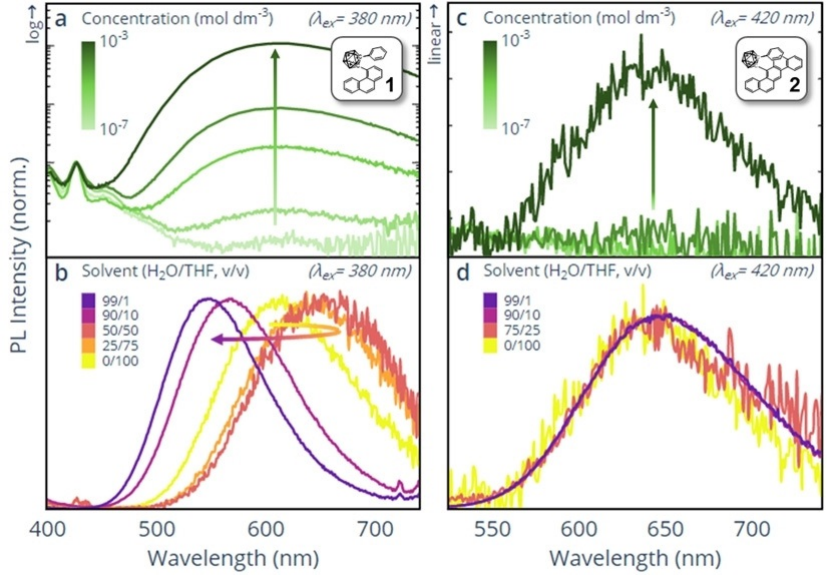
Concentration (THF) and H_2_O/THF v/v solvent dependent PL studies for compound **1** (a) and (b), respectively and compound **2** (c) and (d), respectively. In (a) and (c) the PL spectra are normalised at *λ*=427 nm, and *λ*=537 nm, respectively, whereas for (b) and (d) peak normalisation is used. Concentration =10^−4^ m for (b) and (d), except the 0/100 condition for **2** where the concentration =10^−3^ m owing to its weak luminescence.

Figure 4 a shows that the *λ*_max_ long‐wavelength emission of **1** remains constant with increasing concentration, but its intensity rises relative to the LE. The observation is suggestive of a non‐unimolecular excited state, in which the probability of formation increases with concentration. Furthermore, the emission peak of **1** initially red‐shifts with lower H_2_O ratios, before blue‐shifting with higher H_2_O ratios to match that of the solid state (Figure 4 b). This behaviour strongly resembles our previous reports from a related chrysene compound, which exhibited weak excimer emission in THF solution, and ICT in aggregated states.^[23]^ Accordingly, we propose that the two delocalised species of **1** appearing at *λ*_max_=548 nm and 615 nm are attributable to ICT and excimer emission, respectively. Figure 4 c and 4d demonstrate that **2** is very weakly emissive in THF and in non‐aggregated mixed H_2_O/THF, and exhibits a single emissive species. This species is also responsible for the AIE property of **2** observed in higher H_2_O ratios which, for *o*‐carborane containing compounds of this type, are attributable to an ICT state.[^17^, ^20^, ^27^]

The excimer emission of **1** is unusual, as phenanthrene is not known to form excimers except under exceptional environments.^[53]^ The structure of the excimer has not been elucidated, and we do not discount that the phenanthrene moiety is not electronically involved in this species; however, it is clear that incorporation of *o*‐carborane into this sterically hindered region does induce the excimeric behaviour. Furthermore, the low‐energy nature, particularly when compared to reports of phenanthrene excimers^[54]^ suggests that the carborane is likely to be electronically involved. In good solutions, such as THF, the LE and ICT for both **1** and **2** are either not observed or extremely weak. We believe this is a result of the rigid and perpendicular C_C_−C_C_ geometry favouring ICT formation from the locally excited phenanthrene, and the extremely elongated C_C_−C_C_ bond that enables efficient non‐radiative decay through its vibration. In aggregated states of **1** and **2**, however, this vibration is mechanically inhibited and leads to AIE. Notably, the *φ*_PL_ values in these aggregate states appear lower when compared with values from other similarly hindered *o*‐carboranyl compounds bearing non‐aromatic substituents in the carboranyl C(2) position.[^42^, ^43^] As reported elsewhere, this is likely due to intramolecular π‐interactions between the phenyl group and the aromatic donor, which thereby introduce a non‐radiative decay pathway.^[42]^ It follows that replacement of the phenyl group in **1** and **2**, with a similarly sized non‐aromatic group, may be expected to increase the AIE efficiency of these stimuli‐responsive materials.

In conclusion, we have successfully substituted phenyl‐*o*‐carborane into the most sterically hindered positions of phenanthrene and benzo(k)tetraphene via a BHQ reaction pathway. X‐ray crystallography of the compounds has revealed the C_C_−C_C_ bond to be perpendicular to the aromatic plane, in a geometry aiding ICT. Both the compounds investigated displayed significant distortion in the aromatic structure, with **2** exhibiting the most considerable aromatic deformation parameters of any carborane compound reported to date and among the largest neutral state C_C_−C_C_ bond length for any organo‐substituted carborane. Photoluminescence studies of **1** revealed three distinct emissive peaks which were assigned to LE, ICT, and an unusual excimer species, whilst similar studies of **2** revealed a single ICT emissive peak under all conditions. AIE from the ICT state was demonstrated for both **1** and **2**, aided by the perpendicular orientation of the C_C_−C_C_ bond in the ground state, and their high structural rigidity. These are two new members of the growing family of *o*‐carborane functionalised non‐linear acenes, notable for their highly distorted structures, multiple emissive species, and stimuli‐responsive luminescence.

## Conflict of interest

The authors declare no conflict of interest.

## Supporting information

As a service to our authors and readers, this journal provides supporting information supplied by the authors. Such materials are peer reviewed and may be re‐organized for online delivery, but are not copy‐edited or typeset. Technical support issues arising from supporting information (other than missing files) should be addressed to the authors.

Supplementary
