# Supplementary material for: Highly Deformed o‐Carborane Functionalised Non‐linear Polycyclic Aromatics with Exceptionally Long C−C Bonds
Source: Chemistry. 2020 Dec 23;27(6):1970–5. doi: 10.1002/chem.202004517 (PMC7898797; doi:10.1002/chem.202004517)
Supplement: Supplementary file 1 — Supplementary [file CHEM-27-1970-s001.pdf]

Chemistry–A European Journal

Supporting Information

Highly Deformed *o*-Carborane Functionalised Non-linear Polycyclic Aromatics with Exceptionally Long C–C Bonds

Adam V. Marsh,[a] Mark Little,[a] Nathan J. Cheetham,[b] Matthew J. Dyson,[c] Matthew Bidwell,[a] Andrew J. P. White,[a] Colin N. Warriner,[d] Anthony C. Swain,[d] Iain McCulloch,[e] Paul N. Stavrinou,*[f] and Martin Heeney*[a]

Table of Contents

Experimental Procedures 3
High-Resolution Mass Spectrometry 9
1 9
2 10
NMR 11
1 11
2 13
XRD Refinement Data 15
Aromatic Deformation Parameters 16
Survey of Literature Carborane Compounds 17
Computational Potential Energy Surface Scan 18
Optical Spectra 19
Solvent-Dependent 19
Concentration-Dependent 20
References 21

Experimental Procedures

All solvents, reactants, and reagents were purchased from Acros Organics, Alfa Aesar, Apollo Scientific, Sigma Aldrich, TCI Europe, or VWR, and used without further purification. Reactions were carried out under BOC Pureshield Argon unless otherwise stated. Proton (^{1}H), Boron (^{11}B), and carbon (^{13}C) solution-state NMR spectra were obtained from a Bruker Avance-400 (400 MHz) spectrometer, at room temperature using $CDCl_3$ as the deuterated solvent. Chemical shift data was obtained using residual $CDCl_3$ internal standards (^{1}H: 7.26 ppm; ^{13}C: 77.36 ppm); singlet = s, doublet = d, triplet = t, quarter = q, multiplet = m, broad = br, with combinations for complex multiplets. High resolution mass spectrometry (HRMS) data was obtained from an Agilent HP6890 GC (EI); Dinoex Ultimate 3000 (APCI); and ultrafleXtreme (MALDI-ToF). Infra-red (ATR-IR) spectroscopy measurements were obtained using a PerkinElmer FT-IR Spectrum 100 fitted with an ATR (Ge/Ge) accessory. X-ray crystallographic data was obtained with an Agilent Xcalibur PX Ultra A, at 173 K. Cyclic Voltammetry data was obtained using a Metrohm Autolab PGSTAT101 potentiostat in dry DCM containing 0.1M NBu_4PF_6 with a platinum working electrode, Pt counter electrode and an Ag/AgCl (Ag/Ag^{+}) reference electrode with ferrocene as internal reference.

Density functional theory (DFT) calculations were carried out using B3LYP[1,2] functional and 6-31G(d,p)[3,4] basis set, with no symmetry constraints, in the gas phase. Dihedral scans were obtained using OPT(Z-MAT) optimisation, starting from the calculated global ground state geometry and proceeding with 1° increments around the bond connecting the carborane and the substituent.

Warning: Exposure to polycyclic aromatic hydrocarbon (PAH) containing substances increases the risk of cancer in humans.[5] Replication of these syntheses should be proceeded with caution.

1-(Allyloxy)naphthalene[6]

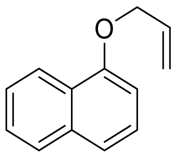

Allyl bromide (17.99 mL, 208 mmol) was added to a suspension of 1-naphthol (15.00 g, 104 mmol) and K_2CO_3 (43.13 g, 312 mmol) in dry acetonitrile (300 mL) and stirred overnight at room temperature. The inorganic components were separated by vacuum filtration and washed with diethyl ether (300 mL). The filtrate was washed with water (5 x 200 mL), brine (2 x 200 mL) and dried over $MgSO_4$. Solvent was removed *in vacuo* yielding the *title compound* as a yellow oil (17.63 g, 95.7 mmol, 92%), which was subsequently used without further purification. ^{1}H NMR (400 MHz, $CDCl_3$) δ 8.45-8.40 (1H, m), 7.91-7.85 (1H, m) 7.62-7.39 (4H, m), 6.86 (1H, d, *J* = 7.8 Hz), 6.30-6.19 (1H, m), 5.62 (1H, dd, *J* = 17.2, 1.5 Hz), 5.41 (1H, dd, *J* = 10.6, 1.5 Hz) 4.76 (2H, d, *J* = 5.1 Hz) ppm. ^{13}C NMR (100 MHz, $CDCl_3$) δ 154.6, 134.9, 133.6, 127.8, 126.7, 126.1, 126.0, 125.5, 122.4, 120.7, 117.6, 106.4, 69.2 ppm. HRMS (EI$^+$) $C_{13}H_{12}O$ requires 184.0888, found 184.0879.

2-Allyl-1-(2,2,2-trichloroacetyl)naphthalene[6]

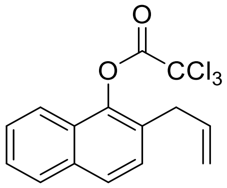

1-(Allyloxy)naphthalene (15.00 g, 81.4 mmol) was heated under argon, with stirring, for 2 h at 210 °C, after which the heating was removed and the brown oil was allowed to cool to room temperature. Dry diethyl ether (300 mL) and pyridine (9.00 mL, 111 mmol) were added, the solution was cooled to 0 °C and trichloroacetyl chloride (12.50 mL, 111 mmol) was added dropwise. The mixture was stirred for 2 h then allowed to warm to room temperature. The brown solution was poured into a slurry of ice-water (200 g) and $NaHCO_3$ (5 g), washed with water (2 x 100 mL), saturated aq. NH_4Cl (3 x 100 mL), water (3 x 100 mL), brine (2 x 100 mL), and dried over $MgSO_4$. Solvent was removed *in vacuo* yielding the *title compound* as an orange solid (22.81 g, 69.2 mmol, 85%), which was subsequently used without further purification. ^{1}H NMR (400 MHz, $CDCl_3$) δ 7.91-7.84 (2H, m), 7.80 (1H, d, *J* = 8.5 Hz), 7.60-7.48 (2H, m) 7.41 (1H, d, *J* = 8.5 Hz) 6.03-5.91 (1H, m), 5.16 (1H, m), 5.13 (1H, m, *J* = 1.5 Hz) 3.52 (2H, m) ppm. ^{13}C NMR (100 MHz, $CDCl_3$) δ 160.7, 143.8, 135.4, 133.9, 128.8, 128.4, 128.0, 127.7, 127.6, 126.6, 120.6, 117.4, 34.6 ppm. HRMS (APCI) $C_{15}H_{11}Cl_3O_2$ requires 328.9897, found 328.9897.

4-Chlorophenanthrene[6]

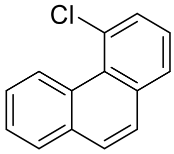

2-Allyl-1-(2,2,2-trichloroacetyl)naphthalene (4.00 g, 12.1 mmol) was dissolved in dry diglyme (4 mL) and the solution was purged with argon for 20 mins. Cuprous chloride (5 mol%, 60 mg, 0.61 mmol) was added and the mixture was purged with argon for a further 20 mins before being heated to reflux for 2 h, or until HCl gas emission ceased. The mixture was cooled, loaded directly onto a flash chromatography column and eluted with 1:9 DCM:Pet. ether. The product fractions were left to slowly evaporate, yielding the *title compound* as a white crystalline solid (1.24 g, 5.83 mmol, 48%). ^{1}H NMR (400 MHz, $CDCl_3$) δ 10.59-10.50 (1H, m), 8.16 (1H, dd, *J* = 7.3, 1.4 Hz), 7.98-7.92 (2H, m), 7.80 (2H, m), 7.70-7.63 (3H, m) ppm. ^{13}C NMR (100 MHz, $CDCl_3$) δ 134.6, 133.6, 133.4, 131.1, 130.2, 128.8, 128.3, 127.8, 127.3, 126.7, 126.6, 126.1, 120.2, 98.1 ppm. HRMS (EI$^+$) $C_{14}H_9Cl$ requires 212.0393, found 212.0402.

4-(Phenylethynyl)phenanthrene

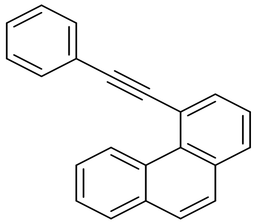

4-Chlorophenanthrene (2.00 g, 9.40 mmol), cesium carbonate (3.06 g, 9.40 mmol), bis(triphenylphosphine)palladium(II) dichloride (10 mol%, 660 mg, 0.94 mmol) tri(cyclohexyl)phosphine (20 mol%, 527 mg, 1.88 mmol) and phenylacetylene (4.13 mL, 37.6 mmol) were added to a flask charged with dry DMF (30 mL), and the mixture was purged with argon before being heated to 110 °C overnight. After cooling to room temperature, the mixture was added directly to a silica plug (Pet. ether), which was eluted first with Pet. ether, then 1:5 DCM:Pet. ether to eluted the crude product. Solvent was removed *in vacuo* and the crude product was subjected to column chromatography Pet. ether eluent, yielding the *title compound* as colourless crystals (893 mg, 3.20 mmol, 34%). ^{1}H NMR (400 MHz, $CDCl_3$) δ 10.40 (1H, dd, *J* = 8.3, 0.8 Hz), 7.98 (1H, dd, *J* = 7.4, 1.4 Hz), 7.91 (2H, td, *J* = 8.3, 1.4 Hz), 7.75 (2H, m), 7.72-7.62 (4H, m), 7.57 (1H, t, *J* = 7.6 Hz), 7.48-7.38 (3H, m) ppm. ^{13}C NMR (100 MHz, $CDCl_3$) δ 135.2, 133.5, 133.4, 131.7, 131.1, 130.1, 130.0, 128.9, 128.7, 128.2, 127.7, 127.2, 126.6, 126.3, 125.9, 124.1, 119.6, 95.2, 92.8 ppm. HRMS (EI^+) $C_{22}H_{14}$ requires 278.1096, found 278.1103.

4-(2-phenyl-1,2-dicarbadodecaboran-1-yl)phenanthrene (1)

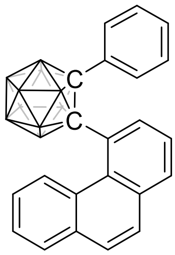

4-(Phenylethynyl)phenanthrene (286 mg, 1.03 mmol) and decaborane (377 mg, 3.09 mmol) were added to a vigorously stirring flask charged with a biphasic mixture of bmim(Cl) (162 mg, 0.93 mmol) and dry toluene (10 mL), and the mixture was heated to reflux for 48 h. After cooling the solvent was removed *in vacuo* and the crude product was subjected to column chromatography with hexane eluent, yielding the *title compound* as bright green crystals (131 mg, 0.33 mmol, 32%). Crystals suitable for X-ray diffraction analysis were grown by slow evaporation of a biphasic solution of DCM and hexane. ^{1}H NMR (400 MHz, $CDCl_3$) δ 8.77 (1H, d, *J* = 8.2 Hz), 8.28 (1H, dd, *J* = 7.7, 1.2 Hz), 7.77 (1H, dd, *J* = 8.0, 1.2 Hz), 7.60 (1H, ddd, *J* = 8.0, 7.0, 1.2 Hz), 7.48 (1H, ddd, *J* = 8.0, 7.0, 1.2 Hz), 7.42 (1H, d, *J* = 8.6 Hz), 7.38 (1H, dd, *J* = 7.7, 1.0 Hz), 7.30 (1H, t, *J* = 7.7 Hz), 7.04 (1H, d, *J* = 8.6 Hz), 6.94 (1H, m), 6.67-6.61 (2H, m), 6.50-6.44 (2H, m), 4.29-1.51 (10H, br) ppm. ^{11}B NMR (128 MHz, $CDCl_3$) δ -0.68, -1.85, -2.60, -3.78, -9.74, -10.90 ppm. ^{13}C NMR (100 MHz, $CDCl_3$) δ 139.6, 133.3, 133.2, 133.0, 132.6, 130.4, 130.2, 129.9, 129.5, 127.8, 127.6, 127.0, 126.9, 126.8, 124.1, 123.4, 90.9, 89.3 ppm. HRMS (EI^+) $C_{22}B_{10}H_{24}$ requires 396.2916, found 396.2908. ATR-IR (*v* cm^{-1}) 3061, 2593 (B-H) br, 2556 (B-H) br, 2210, 2166, 2051, 1978, 1919, 1604, 1492, 1446, 1257, 1332, 1074, 1019, 1003, 880, 792, 778, 744, 718, 705, 685.

2-Bromo-1-tetralone[6]

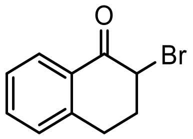

Note: the target compound is a lachrymator and should be treated with caution.

To a stirring solution of 1-tetralone (18.20 mL, 137 mmol) in dry diethyl ether (100 mL) cooled to 0 °C, was added bromine (7.02 mL, 137 mmol) dropwise over 5 mins, and the mixture was stirred for a further 30 mins. Ice water (100 mL) was added to the reaction, followed by sat. sodium sulfite solution (100 mL). The organic layer was separated, washed with water (3 x 100 mL), brine (100 mL), dried over $MgSO_4$. Solvent was removed *in vacuo* to yield the *title compound* as a brown oil (28.37 g, 126 mmol, 92%) which was subsequently used without further purification. ^{1}H NMR (400 MHz, $CDCl_3$) δ 8.09 (1H, dd, *J* = 8.0, 1.4 Hz), 7.52 (1H, td, *J* = 7.6, 1.4 Hz), 7.35 (1H, t, *J* = 8.0 Hz), 7.28 (1H, d, *J* = 7.6 Hz), 4.73 (1H, dd, *J* = 5.1, 3.8 Hz), 3.32 (1H, ddd, *J* = 17.1, 9.8, 4.8 Hz), 2.92 (1H, dt, *J* = 17.1, 4.4, Hz), 2.59-2.41 (2H, m) ppm. ^{13}C NMR (100 MHz, $CDCl_3$) δ 190.9, 143.3, 134.4, 130.3, 129.1, 129.0, 127.4, 50.8, 32.3, 26.5 ppm. HRMS (EI$^+$) $C_{10}H_9BrO$ requires 223.9837, found 223.9824.

2-(Naphthalene-1’-yloxy)-1-tetralone[6]

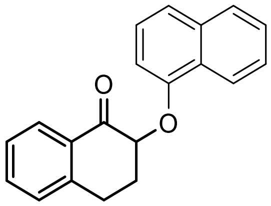

To a stirring suspension of 1-naphthol (14.79 g, 103 mmol) and K_2CO_3 (28.37 g, 205 mmol) in dry acetone (300 mL) was added 2-bromo-1-tetralone (23.12 g, 103 mmol) at room temperature, in one portion, and the mixture was stirred overnight. The inorganic material was removed by vacuum filtration, washed with acetone, and the organic fractions were concentrated *in vacuo*. The brown residue was dissolved in diethyl ether (300 mL), washed with NaOH solution (1.0 M, 3 x 100 mL), water (3 x 100 mL), brine (100 mL) and dried over $MgSO_4$. Solvent was removed in *vacuo* to afford the *title compound* as a light brown solid (19.31 g, 67.0 mmol, 65%). ^{1}H NMR (400 MHz, $CDCl_3$) δ 8.37-8.30 (1H, m), 8.09 (1H, dd, *J* = 8.0, 1.0 Hz), 7.85-7.77 (1H, m), 7.58-7.44 (4H, m), 7.41-7.34 (2H, m), 7.32 (1H, d, *J* = 7.6 Hz), 6.98 (1H, d, *J* = 7.6 Hz), 5.15 (1H, dd, *J* = 10.4, 5.2 Hz), 3.35-3.13 (2H, m), 2.70-2.54 (2H, m) ppm. ^{13}C NMR (100 MHz, $CDCl_3$) δ 195.0, 154.3, 143.6, 135.0, 134.2, 132.3, 129.0, 128.4, 127.8, 127.3, 126.7, 122.6, 121.6, 107.8, 79.7, 30.4, 27.8 ppm. HRMS (EI$^+$) $C_{20}H_{16}O_2$ requires 288.1150, found 288.1147.

2’-(Naphthalene-1’-yloxy)-1-methylene-2,3,4-trihydronaphthalene[6]

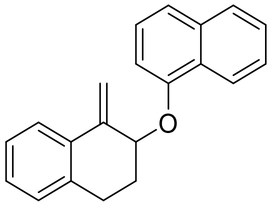

To a vigorously stirring suspension of 2-(Naphthalene-1’-yloxy)-1-tetralone (15.00 g, 52.0 mmol) in dry diethyl ether (200 mL) was added a ylide solution freshly prepared from the addition of methyltriphenylphosphonium bromide (28.17 g, 78.9 mmol) and KO^{t}Bu (8.85 g, 78.9 mmol) to a solution of dry diethyl ether (200 mL) at 0 °C, which had been left stirring for 30 mins. The resulting bright yellow mixture was left stirring overnight, then the inorganic material was removed via suction filtration through celite. The filtration was concentrated *in vacuo* and dry loaded onto a silica plug which was eluted with Pet. ether until a yellow colour appeared in the filtrate, after which eluent was changed to 1:9 DCM:Pet. ether. The yellow filtrate fractions were combined and left to evaporate slowly yielding the *title compound* as bright yellow crystals (7.89 g, 27.6 mmol, 53%). ^{1}H NMR (400 MHz, $CDCl_3$) δ 8.21 (1H, d, J = 8.2 Hz), 7.79 (1H, d, J = 8.0 Hz), 7.64 (1H, dd, J = 8.0, 1.4 Hz), 7.49-7.29 (4H, m), 7.26-7.16 (3H, m), 6.97 (1H, d, J = 7.5 Hz), 5.65 (1H, s), 5.36, (1H, s), 5.24 (1H, dd, *J* = 7.8, 3.2 Hz), 3.21 (1H, dt, *J* = 17.0, 6.4 Hz), 3.0 (1H, dt, *J* = 17.0, 6.4 Hz), 2.44-2.27 (2H, m) ppm. ^{13}C NMR (100 MHz, $CDCl_3$) δ 154.1, 143.3, 136.2, 135.0, 134.2, 129.2, 128.4, 127.8, 126.9, 126.6, 126.6, 126.1, 125.5, 125.5, 122.7, 120.9, 110.4, 107.9, 77.7, 29.7, 27.3 ppm. HRMS (EI$^+$) $C_{21}H_{18}O_2$ requires 286.1358, found 286.1360.

2'-((3,4-Dihydronaphthalen-1-yl)methyl)naphthalen-1'-trichloroacetate[6]

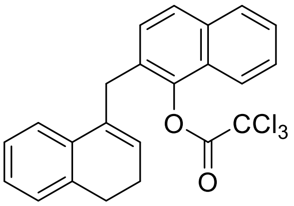

A solution of 2'-(Naphthalene-1'-yloxy)-1-methylene-2,3,4-trihydronaphthalene (19.00 g, 66.3 mmol) in pyridine (18.7 mL, 232 mmol) was heated in an inert atmosphere, with stirring, for 2 h at 115 °C after which the solution was allowed to cool to room temperature. The residue was dissolved with dry diethyl ether (300 mL), cooled to 0 °C and trichloroacetyl chloride (8.84 mL, 79.0 mmol) was added dropwise. The mixture was stirred for 2 h then allowed to warm to room temperature. The brown solution was poured into a slurry of ice-water (200 g) and $NaHCO_3$ (5 g), washed with water (2 x 100 mL), saturated aq. NH_4Cl (3 x 100 mL), water (3 x 100 mL), brine (2 x 100 mL), and dried over $MgSO_4$. Solvent was removed *in vacuo* yielding the *title compound* as a brown solid (23.5 g, 54.4 mmol, 82%) which was subsequently used without further purification. ^{1}H NMR (400 MHz, $CDCl_3$) δ 7.94 (1H, d, *J* = 8.4 Hz), 7.89 (1H, d, *J* = 8.0 Hz), 7.76 (1H, d, *J* = 8.4 Hz), 7.60 (1H, t, *J* = 7.6 Hz), 7.54 (1H, t, *J* = 7.6 Hz), 7.42 (1H, d, *J* = 8.4 Hz), 7.28-7.10 (4H, m), 5.85 (1H, t, *J* = 4.0 Hz) 3.94 (2H, s), 2.84 (2H, t, *J* = 7.8 Hz), 2.40-2.29 (2H, m) ppm. ^{13}C NMR (100 MHz, $CDCl_3$) δ 160.6, 144.0, 136.8, 134.9, 133.8, 133.6, 128.9 128.9, 128.8, 128.4, 127.9, 127.7, 127.6, 127.6, 127.3, 126.8, 126.6, 123.1, 120.5, 90.1, 33.3, 28.5, 23.6 ppm. HRMS (EI^+) $C_{23}H_{17}Cl_3O_2$ requires 430.0294, found 430.0309.

7-Chloro-5,6-dihydrobenzo[*k*]tetraphene[6]

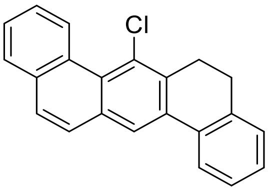

2'-((3,4-Dihydronaphthalen-1-yl)methyl)naphthalen-1'-trichloroacetate (4.00, 9.26 mmol) was dissolved in dry diglyme (5 mL) and the solution was purged with argon for 20 mins. Cuprous chloride (5 mol%, 46 mg, 0.463 mmol) was added and the mixture was purged for a further 10 mins before being heated to reflux for 2 h, or until HCl gas emission ceased. After cooling to room temperature, the crude mixture was subjected to column chromatography using 1:9 DCM:Pet. ether, and the *title compound* was isolated as a beige solid (2.36 g, 7.50 mmol, 81%). ^{1}H NMR (400 MHz, $CDCl_3$) δ 9.92 (1H, d, *J* = 8.1, 1.8 Hz), 8.18 (1H, s), 7.93 (1H, d, *J* = 7.6 Hz), 7.91-7.87 (1H, m), 7.72 (2H, s), 7.68-7.60 (2H, m), 7.42-7.34 (1H, m), 7.34-7.31 (2H, m), 3.38 (2H, dd, *J* = 8.6, 5.7 Hz), 3.00 (2H, dd, *J* = 8.6, 5.7 Hz) ppm. ^{13}C NMR (100 MHz, $CDCl_3$) δ 137.9, 136.5, 134.3, 134.1, 134.0, 133.8, 131.0, 130.0, 128.9, 128.5, 128.4, 128.3, 128.0, 127.6, 127.5, 127.5, 127.0, 126.0, 124.9, 123.1, 29.0, 27.4 ppm. HRMS (EI^+) $C_{22}H_{15}Cl$ requires 314.0862, found 314.0857. The two singlet proton resonances at 7.72 are attributed to the C(12)-H and C(13)-H hydrogens which are magnetically equivalent.[6]

7-Chlorobenzo[*k*]tetraphene[6]

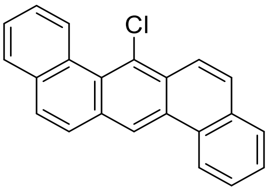

2,3-Dichloro-5,6-dicyano-1,4-benoquinone (DDQ) (10.0 g, 44.0 mmol) was added to a stirring solution of 7-chloro-5,6-dihydrobenzo[*k*]tetraphene (6.00 g, 19.1 mmol) in dry dichlorobenzene (150 mL) and the mixture was heated to 150 °C for 1 h before being allowed to cool to room temperature. Methanol (300 mL) was added and the beige precipitate formed was collected via vacuum filtration, washed with water until the filtrate turned clear, then methanol. Drying yielded the *title compound* as light-brown microcrystals (5.32 g, 39.2 mmol, 89%). ^{1}H NMR (400 MHz, $CDCl_3$) δ 10.00-9.95 (1H, m), 9.07 (1H, s), 8.84 (1H, dd, *J* = 8.2, 0.6 Hz), 8.63 (1H, dd, *J* = 9.6, 0.6 Hz), 7.97-7.84 (4H, m), 7.78-7.64 (5H, m) ppm. ^{13}C NMR (100 MHz, $CDCl_3$) δ 134.2, 132.8, 131.9, 130.1, 130.1, 130.0, 129.8, 128.9, 128.9, 128.6, 128.0, 127.9, 127.7, 127.6, 127.0, 127.0 126.2, 124.4, 123.5, 122.2 ppm. HRMS (EI^+) $C_{22}H_{13}Cl$ requires 312.0706, found 312.0714.

7-(Phenylethynyl)benzo[*k*]tetraphene

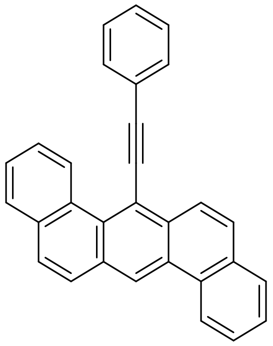

7-Chlorobenzo[*k*]tetraphene (500 mg, 1.60 mmol), cesium carbonate (520 mg, 1.60 mmol), bis(triphenylphosphine)palladium(II) dichloride (10 mol%, 113 mg, 0.16 mmol) tri(cyclohexyl)phosphine (20 mol%, 90 mg, 0.32 mmol) and phenylacetylene (0.71 mL, 4.17 mmol) were added to a flask charged with dry DMF (7 mL), and the mixture was purged with argon before being heated to 110 °C overnight. After cooling to room temperature, the mixture was added directly to a silica plug (Pet. ether), which was eluted first with Pet. ether, then 1:5 DCM:Pet. ether to elute the crude product. Solvent was removed *in vacuo* and the crude product was subjected to column chromatography Pet. ether eluent, yielding the *title compound* as light-orange crystals (200 mg, 0.53 mmol, 33%). Crystals suitable for X-ray diffraction analysis were grown by evaporation from a biphasic solution of DCM and hexane. ^{1}H NMR (400 MHz, $CDCl_3$) δ 10.53 (1H, d, J = 8.0 Hz), 9.10 (1H, s), 8.84 (1H, d, J = 8.0 Hz), 8.80 (1H, d, J = 9.3 Hz), 7.98-7.79 (6H, m), 7.77-7.64 (5H, m), 7.54-7.43 (3H, m) ppm. ^{13}C NMR (100 MHz, $CDCl_3$) δ 133.8, 133.5, 132.1, 131.7, 131.5, 130.4, 130.1, 129.0, 129.0, 128.9, 128.8, 128.7, 128.3, 128.3 127.7, 127.6, 127.6, 127.3, 126.3, 125.8, 124.2 123.9, 123.3, 116.9, 101.9, 91.0 ppm. HRMS (EI^+) $C_{30}H_{18}$ requires 378.1409, found 378.1415.

7-(2-Phenyl-1,2-dicarbadodecaboran-1-yl)benzo[*k*]tetraphene (2)

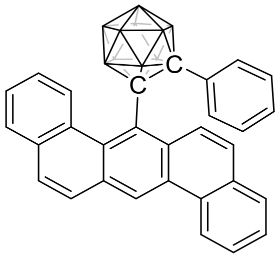

Decaborane (237 mg, 1.94 mmol) was stirred into dry acetonitrile (0.4 mL) and heated to 60 °C for 2 h, after which the mixture was cooled and diluted with dry toluene (10 mL). 7-(Phenylethynyl)benzo[*k*]tetraphene (221 mg, 0.58 mmol) and $AgNO_3$ (9 mg, 0.051 mmol) were added, and the mixture was heated to reflux for 48 h, after which solvent was removed *in vacuo*, and the residue was subjected to column chromatography with hexane eluent. Recrystallization by evaporation of the column fractions product yielded the title compound as red crystals (61 mg, 0.12 mmol, 21%). Crystals suitable for X-ray diffraction analysis were grown by slow evaporation of a biphasic solution of DCM and hexane. ^{1}H NMR (400 MHz, $CDCl_3$) δ 9.23 (1H, d, J = 9.6 Hz), 8.58-8.52 (1H, m), 8.52 (1H, d, J = 8.0 Hz), 8.31 (1H, s), 7.96-7.90 (1H, m), 7.87 (1H, d, J = 9.6 Hz), 7.82 (1H, dd, J = 8.0, 1.1 Hz), 7.70-7.63 (3H, m), 7.53 (1H, ddd, J = 8.0, 7.6, 1.1 Hz), 7.50 (1H, d, J = 8.6 Hz), 7.10 (1H, m), 7.08 (1H, d, J = 8.6 Hz), 6.64 (2H, t, J = 7.6 Hz), 6.16 (2H, d, J = 7.6 Hz) 4.46-1.11 (10H, br) ppm. ^{11}B NMR (128 MHz, $CDCl_3$) δ 0.61, -0.47, -1.87, -3.10, -9.57 ppm. ^{13}C NMR (100 MHz, $CDCl_3$) δ 137.1, 136.1, 134.1, 131.8, 131.2, 131.1, 130.8, 130.7, 130.6, 130.6, 130.0, 129.9, 128.9, 128.8, 128.7, 127.8, 127.7, 127.6, 127.0, 126.7, 126.5, 124.9, 124.7, 123.9, 123.7, 122.7, 96.9, 92.2 ppm. HRMS (MALDI-ToF) $C_{30}B_{10}H_{28}$ requires 497.3, found 497.3. ATR-IR (v cm^{-1}) 3784, 2549 (B-H) br, 2218, 2040, 1953, 1602, 1486, 1446, 1258, 1147, 1068, 1029, 884, 825, 795, 749, 689, 663.

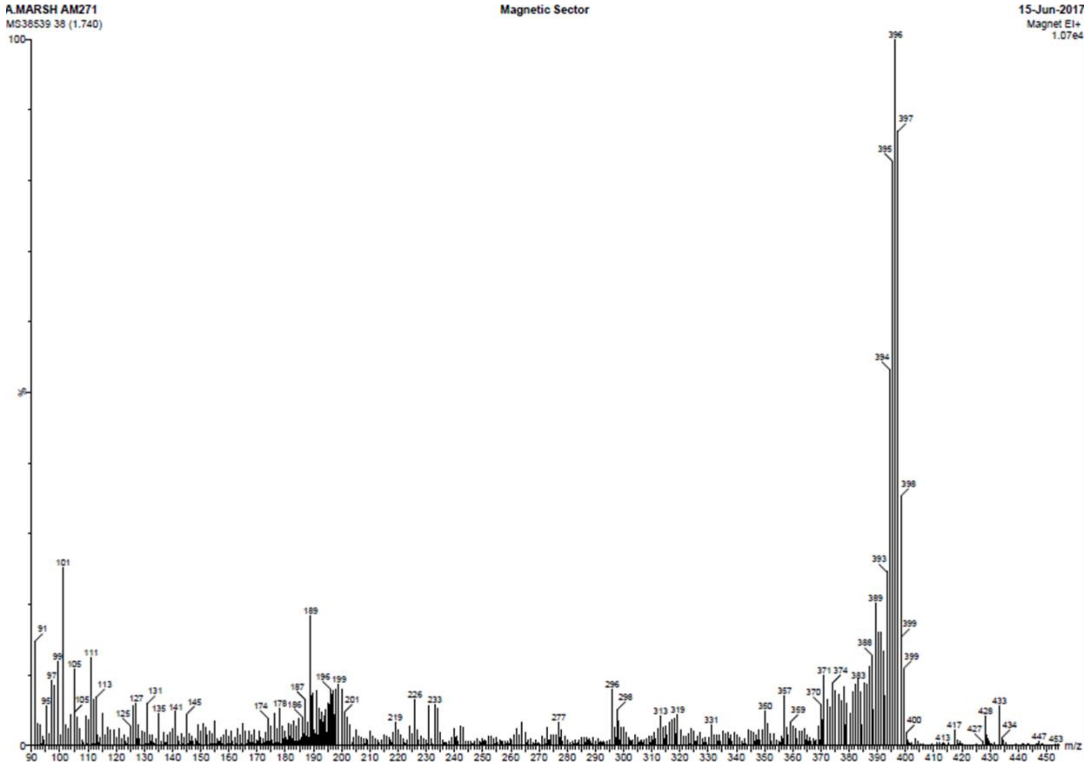

Elemental Composition Report Page 1

Single Mass Analysis (displaying only valid results)
Tolerance = 10.0 PPM / DBE: min = -1.5, max = 50.0
Selected filters: None

Monoisotopic Mass, Odd and Even Electron Ions
328 formula(e) evaluated with 2 results within limits (up to 30 closest results for each mass)
Elements Used:
C: 22-22 H: 0-150 I: 0-2 10B: 0-10 11B: 0-10

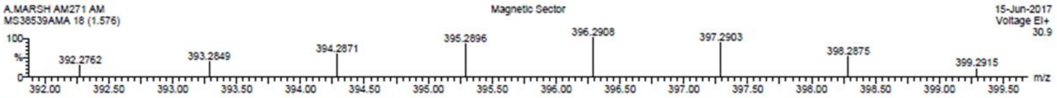

Minimum:				-1.5		
Maximum:		7.0	10.0	50.0		
Mass	Calc. Mass	mDa	PPM	DBE	i-FIT	Formula
396.2908	396.2916	-0.8	-2.0	18.5	1324.2	C22 H20 10B9 11B2
	396.2881	2.7	6.8	16.0	0.1	C22 H24 10B2 11B8

Figure S1. High Resolution Mass Spectrometry data of **1**.

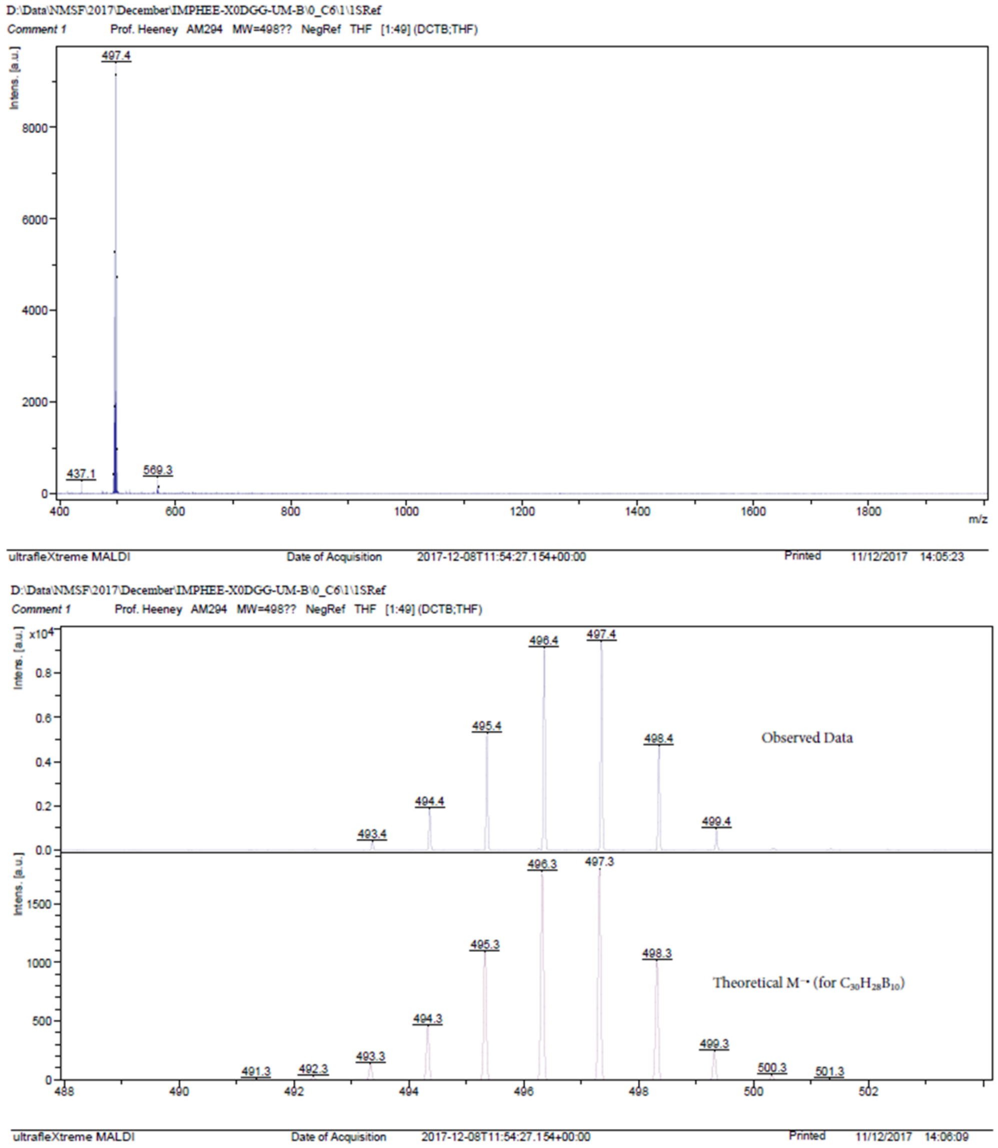

Figure S2. High Resolution Mass Spectrometry data of **2**.

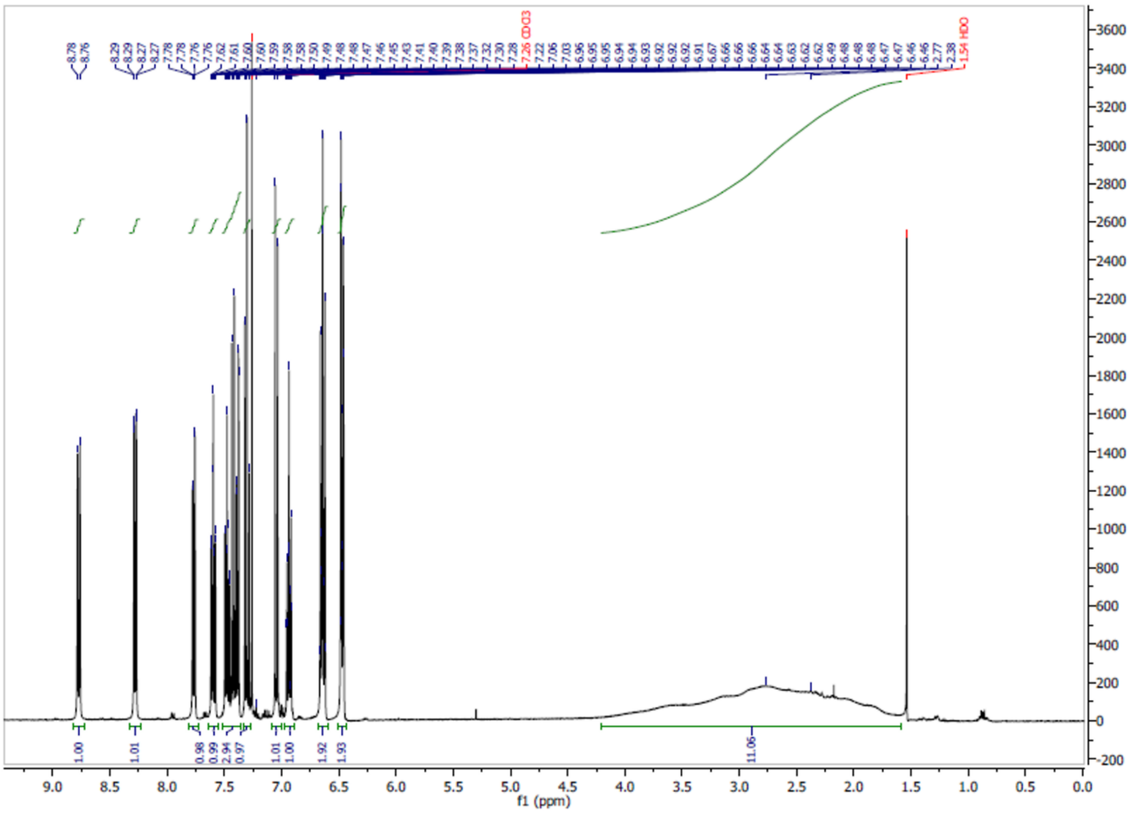

Figure S3. ^{1}H NMR Spectrum of **1**.

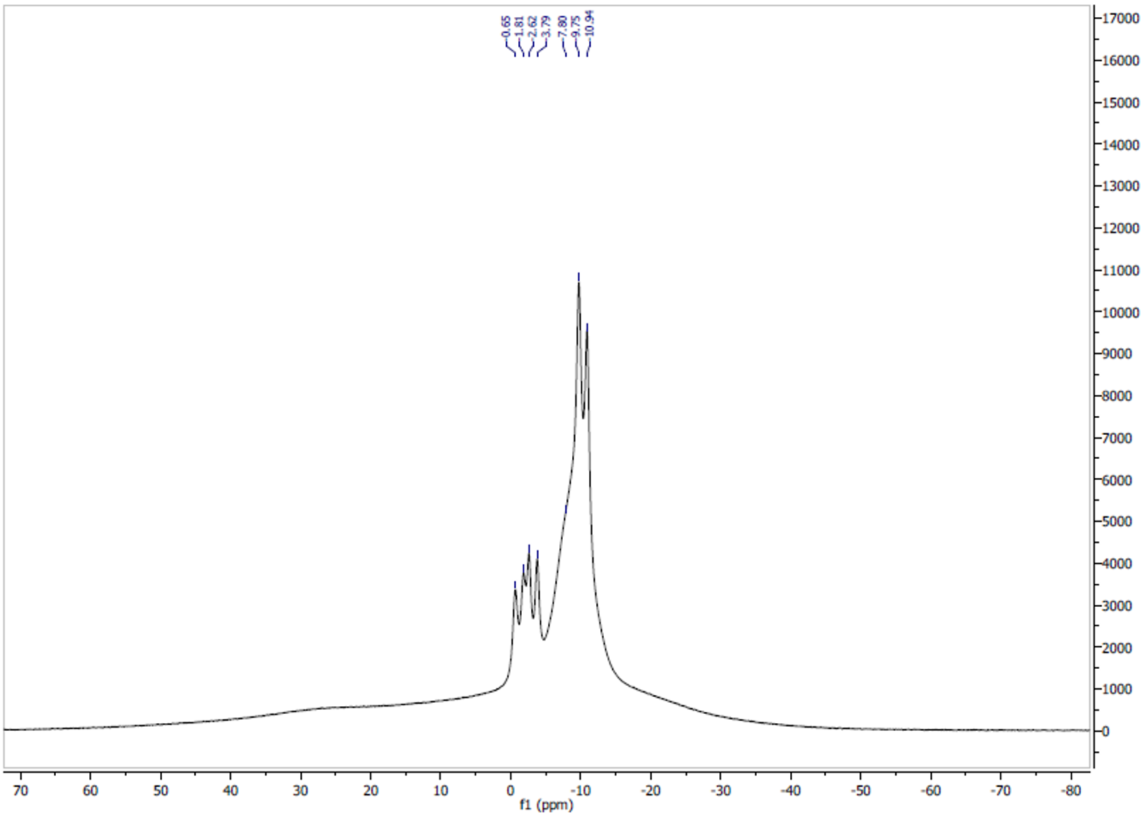

Figure S4. ^{11}B NMR Spectrum of **1**.

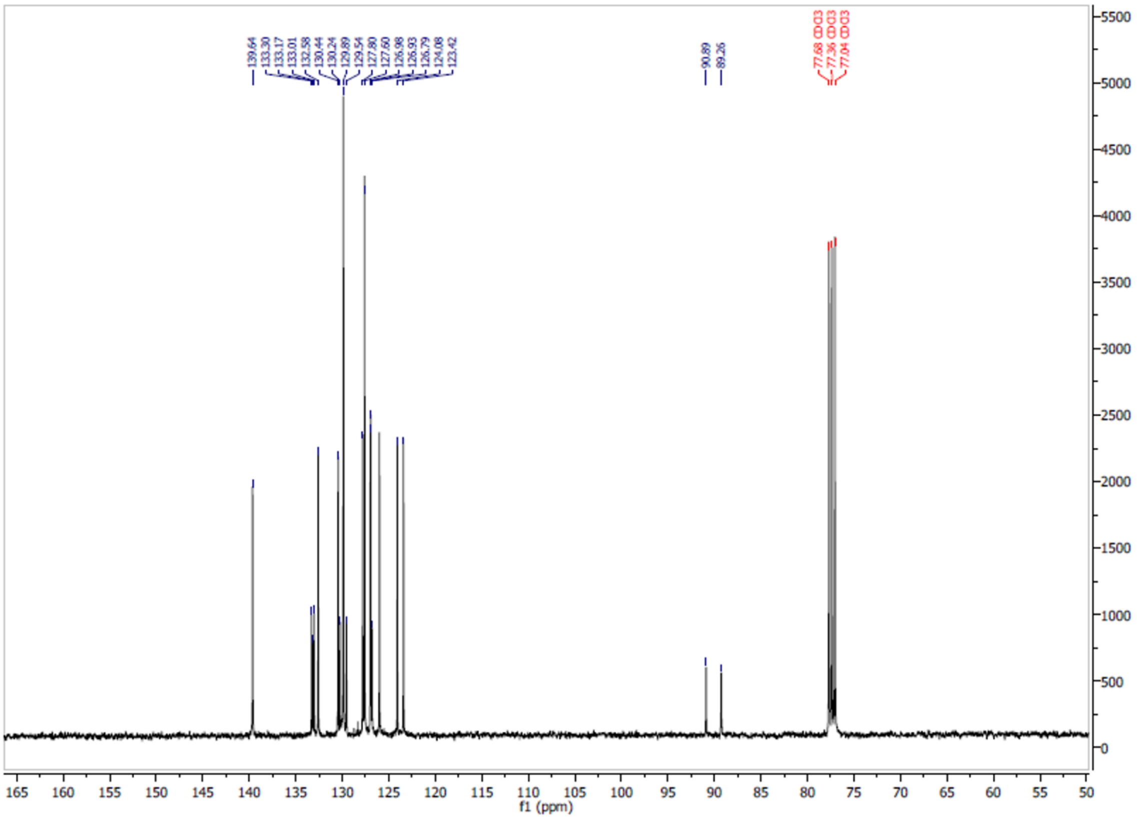

Figure S5. ^{13}C NMR Spectrum of **1**.

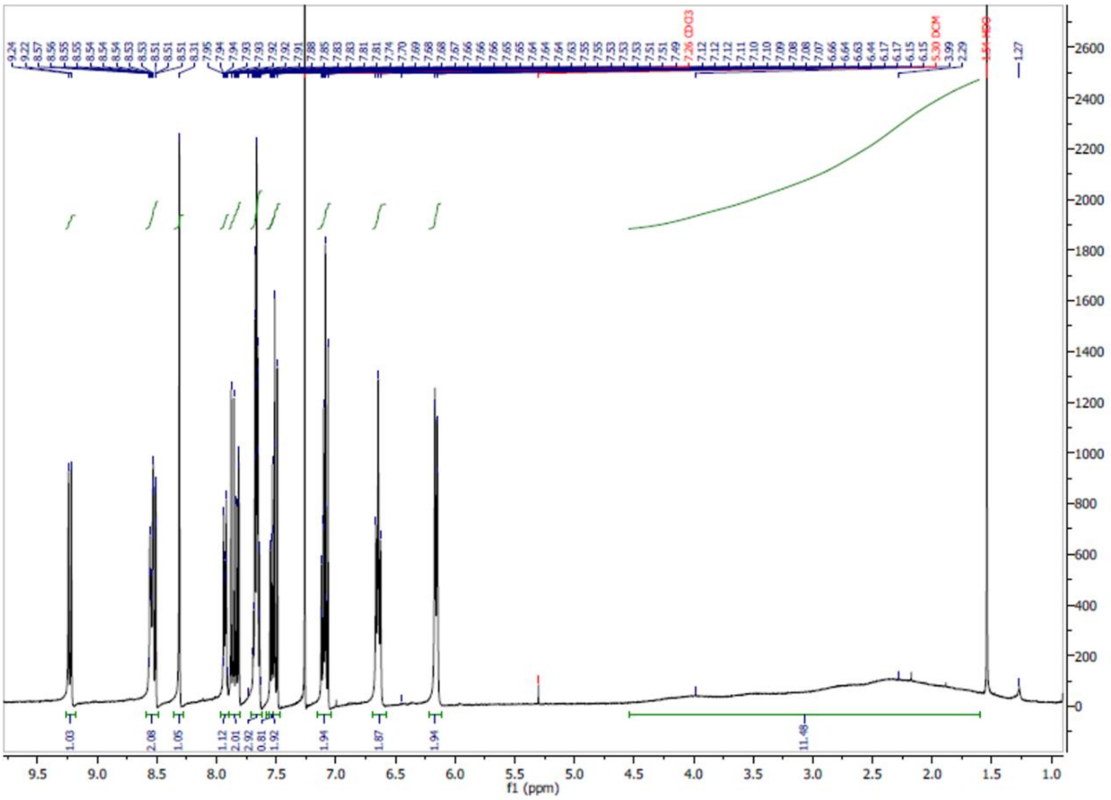

Figure S6. ^{1}H NMR Spectrum of **2**.

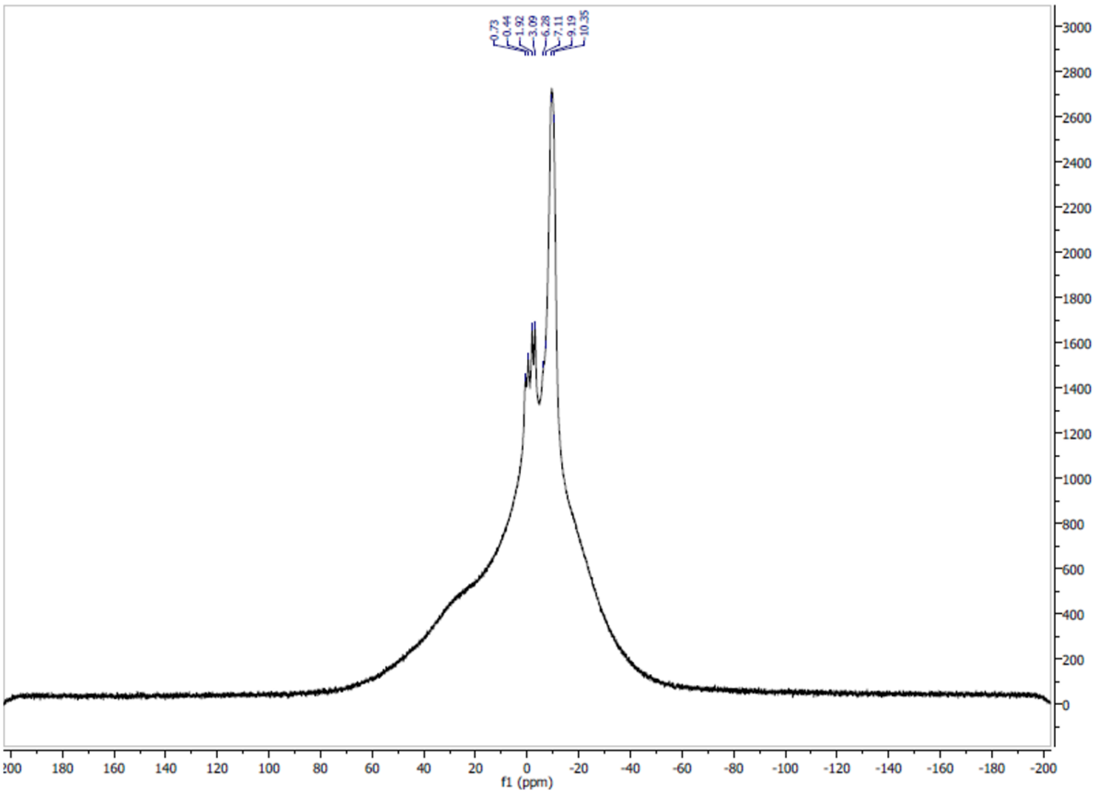

Figure S7. ^{11}B NMR Spectrum of **2**.

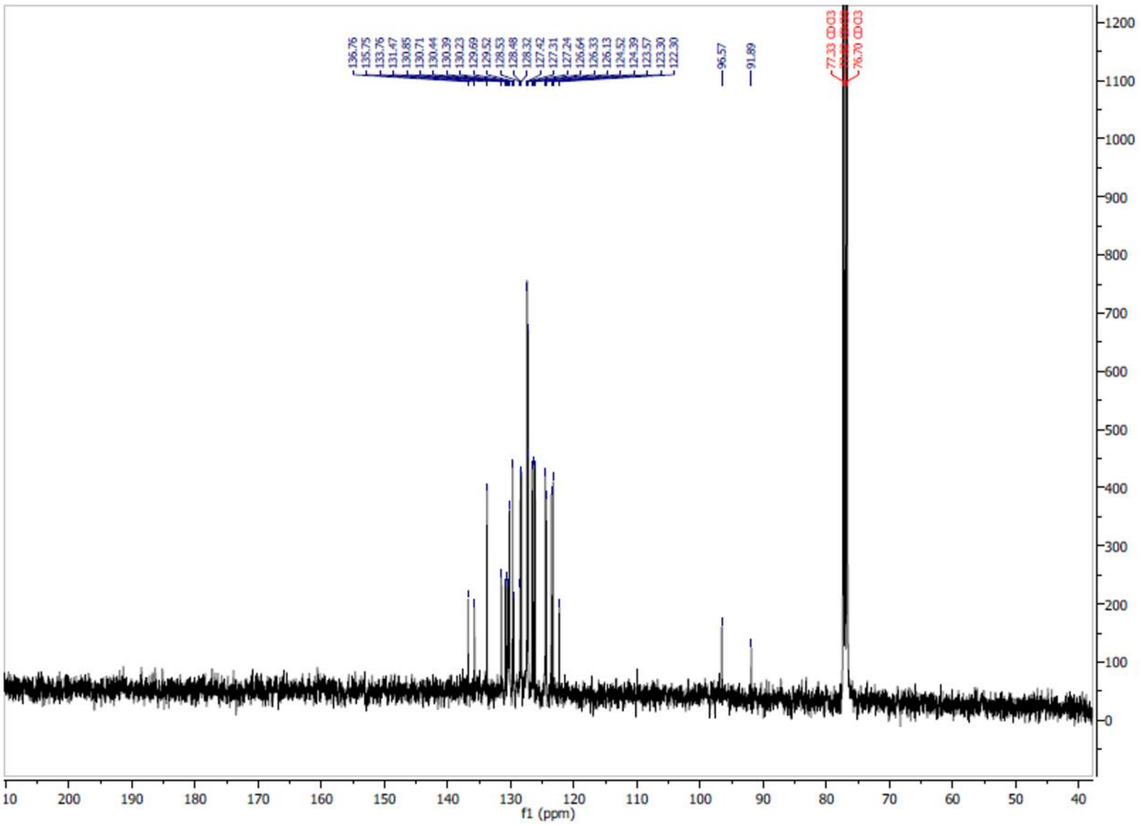

Figure S8. ^{13}C NMR Spectrum of **2**.

Crystallography Data

The X-ray crystal structure of 1

Crystal data for **1**: $C_{22}H_{24}B_{10}$, M = 396.51, triclinic, P-1 (no. 2), a = 10.9407(5), b = 13.8980(6), c = 16.4885(8) Å, α = 109.144(4), β = 91.991(4), γ = 110.785(4)°, V = 2181.68(19) Å^3, Z = 4 [two independent molecules], D_c = 1.207 g cm^{-3}, μ(Mo-Kα) = 0.061 mm^{-1}, T = 173 K, colourless blocks, Agilent Xcalibur 3 E diffractometer; 8575 independent measured reflections (R_{int} = 0.0165), F^2 refinement,[7,8] R_1(obs) = 0.0480, wR_2(all) = 0.1250, 6677 independent observed absorption-corrected reflections [$|F_o| > 4\sigma(|F_o|)$, completeness to θ_{full}(25.2°) = 98.3%], 578 parameters. CCDC 2026711. The structure of **1** was found to crystallise with two independent molecules, **1**-(i) and **1**-(ii), in the asymmetric unit.

The X-ray crystal structure of 2

Crystal data for **2**: $C_{30}H_{28}B_{10}$, M = 496.62, monoclinic $P2_1/c$ (no. 14), a = 20.6054(7), b = 12.8214(4), c = 20.2210(8) Å, β = 96.661(3)°, V = 5306.1(3) Å^3, Z = 8 [two independent molecules], D_c = 1.243 g cm^{-3}, μ(Mo-Kα) = 0.065 mm^{-1}, T = 173 K, orange blocks, Agilent Xcalibur 3 E diffractometer; 10564 independent measured reflections (R_{int} = 0.0269), F^2 refinement,[7,8] R_1(obs) = 0.0653, wR_2(all) = 0.1881, 6998 independent observed absorption-corrected reflections [$|F_o| > 4\sigma(|F_o|)$, completeness to θ_{full}(25.2°) = 98.6%], 721 parameters. CCDC 2026712. The structure of **2** was found to crystallise with two independent molecules, **2**-(i) and **2**-(ii), in the asymmetric unit.

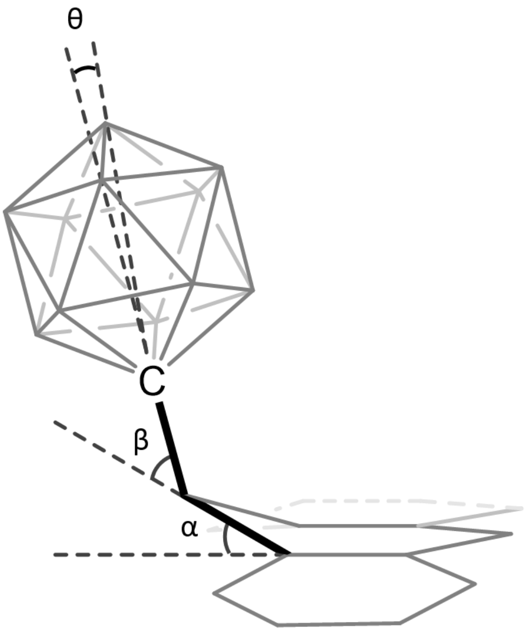

Figure S9. Depiction of the aromatic deformation parameters α and β, as defined in [9], with parameter θ, describing the deviation from the ideal carborane axis of rotation angle of 180°.

Table S3. Survey of aromatic deformation parameters and C_C-C_C bond lengths for literature compounds.

Compound	α + β (°)	C_C-C_C Bond Length (Å)
**2 (i)**	**37.3**	**1.830**
**2 (ii)**	**35.8**	**1.827**
4,10-bis(2-phenyl-1,2-*o*-carborane)chrysene[10]a	33.0	1.767
**1 (i)**	**28.8**	**1.759**
9,10-Bis(2-phenyl-*o*-carborane)anthracene[11]	27.9	-[b]
5,12-Bis(2-phenyl-*o*-carborane)tetracene[12]	27.4	-[b]
**1 (ii)**	**25.7**	**1.769**
9-(2-phenyl-*o*-carborane)anthracene[13]	23.7	1.820
1-(*o*-carborane)anthracene[14]	20.8	1.664
4,8-Bis(2-(4-t-butyl)-phenyl-*o*-carborane)benzodithiophene[15]a	20.8	1.756
9-(2-trimethylsilyl-*o*-carborane)anthracene[13]	18.0	1.778
9-(2-methyl-*o*-carborane)anthracene[13]	17.9	1.758
4,8-Bis(2-phenyl-*o*-carborane)benzodithiophene*[15]	17.6	1.817
1,2-(1-dinapthyl)-*o*-carborane[16]	7.22	1.793

[a]Averaged values for both carboranes. [b]Data not available.

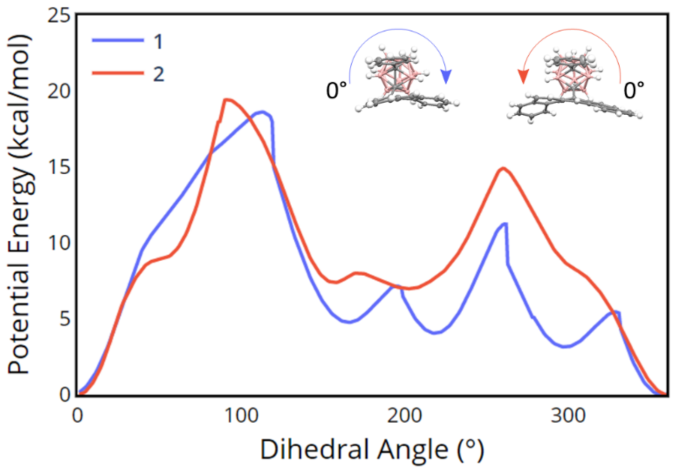

Figure S10. DFT (B3LYP/6-31G(d,p)) calculated potential energies of 1 and 2, as a function of dihedral rotation about the *o*-carborane/aryl bond. Rotation of the carborane occurs in 1° increments in the directions depicted. Energies normalised to the ground state at 0°.

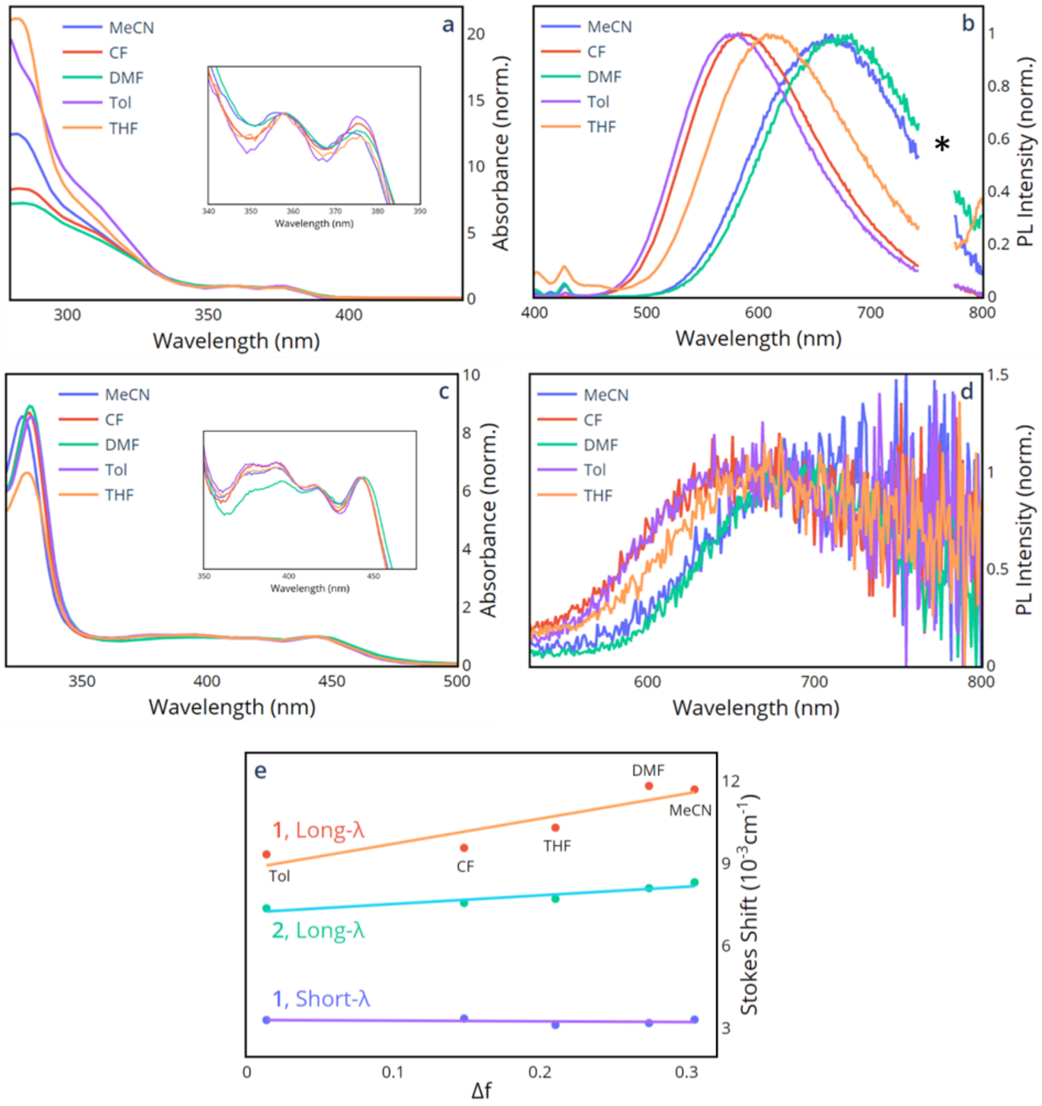

Figure S11. UV-Vis and PL of **1** ((a) and (c), respectively), and **2** ((b) and (d), respectively); (e) Lippert-Mataga[17,18] plot of the short and long-wavelength emissions of **1**, and long-wavelength emission of **2**. The low signal to noise ratio of (d) is a function of the extremely weak emission of **2** in good solutions and, accordingly, λ_{max} values for the Stokes shift calculations are estimates. Concentration ≈ 10^{-4} M, except (d) where concentration ≈ 10^{-3} M owing to the poor luminescence properties of **2**. Asterisk denotes removal of the excitation harmonic.

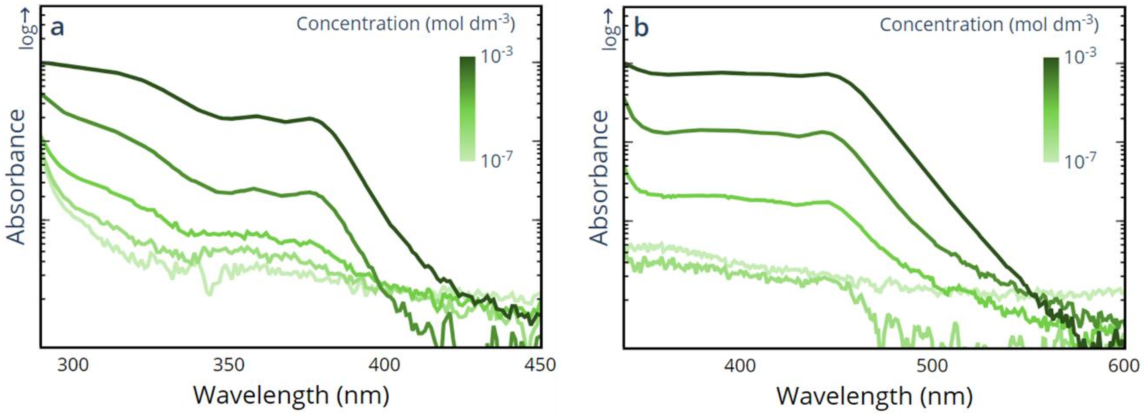

Figure S12. Concentration-dependent UV-Vis of **1** (a), and **2** (b).

References

[1] A. D. Becke, *J. Chem. Phys.* **1993**, *98*, 5648–5652.
[2] C. Lee, W. Yang, R. G. Parr, *Phys. Rev. B* **1988**, *37*, 785–789.
[3] G. A. Petersson, M. A. Al-Laham, *J. Chem. Phys.* **1991**, *94*, 6081–6090.
[4] G. A. Petersson, A. Bennett, T. G. Tensfeldt, M. A. Al-Laham, W. A. Shirley, J. Mantzaris, *J. Chem. Phys.* **1988**, *89*, 2193–2218.
[5] Boström Carl-Elis, Gerde Per, Hanberg Annika, Jernström Bengt, Johansson Christer, Kyrklund Titus, Rannug Agneta, Törnqvist Margareta, Victorin Katarina, Westerholm Roger, *Environmental Health Perspectives* **2002**, *110*, 451–488.
[6] M. Little, The Synthesis of Novel Polycyclic Aromatic Hydrocarbons: The Search for Organic Semiconductor Materials, PhD Thesis, University of Manchester, **2014**.
[7] Bruker AXS, *SHELXTL v5.1*, Madison, WI, **1998**.
[8] G. M. Sheldrick, *Acta Cryst. A* **2015**, *71*, 3–8.
[9] Y. Tobe, S. Saiki, N. Utsumi, T. Kusumoto, H. Ishii, K. Kakiuchi, K. Kobiro, K. Naemura, *J. Am. Chem. Soc.* **1996**, *118*, 9488–9497.
[10] A. V. Marsh, N. J. Cheetham, M. Little, M. Dyson, A. J. P. White, P. Beavis, C. N. Warriner, A. C. Swain, P. N. Stavrinou, M. Heeney, *Angew. Chem. Int. Ed.* **2018**, *57*, 10640–10645.
[11] H. Naito, Y. Morisaki, Y. Chujo, *Angew. Chem. Int. Ed.* **2015**, *54*, 5084–5087.
[12] H. Naito, K. Nishino, Y. Morisaki, K. Tanaka, Y. Chujo, *Chem. Asian J.* **2017**, *12*, 2134–2138.
[13] H. Naito, K. Nishino, Y. Morisaki, K. Tanaka, Y. Chujo, *J. Mater. Chem. C* **2017**, *5*, 10047–10054.
[14] H. Naito, K. Nishino, Y. Morisaki, K. Tanaka, Y. Chujo, *Angew. Chem. Int. Ed.* **2017**, *56*, 254–259.
[15] K. Nishino, K. Uemura, K. Tanaka, Y. Morisaki, Y. Chujo, *Eur. J. Org. Chem.* **2018**, *2018*, 1507–1512.
[16] K.-R. Wee, Y.-J. Cho, J. K. Song, S. O. Kang, *Angew. Chem. Int. Ed.* **2013**, *52*, 9682–9685.
[17] E. Lippert, *Electrochemistry* **1957**, *61*, 962–975.
[18] N. Mataga, Y. Kaifu, M. Koizumi, *Bull. Chem. Soc. Jpn.* **1956**, *29*, 465–470.

Author Contributions

A. V. Marsh – Investigation, data curation, writing of original draft (lead)
M. Little – Synthesis of PAHs (supporting)
N. J. Cheetham – Optical Studies (supporting)
M. J. Dyson – Optical Studies (supporting)
M. Bidwell – Qualitative Data, Optical Studies (supporting)
A. J. P. White – Crystallography (supporting)
C. N. Warriner – Funding Acquisition (supporting)
A. C. Swain – Funding Acquisition (supporting)
I. McCulloch – Project support, supervisor of ML and MB (supporting)
P. Stavrinou – Project support, optical study analysis, supervisor of NJC and MJD (Corresponding)
M. Heeney – Principle Investigator (Corresponding)
